# Supplementary material for: Knock-Out of the Five Lysyl-Oxidase Family Genes Enables Identification of Lysyl-Oxidase Pro-Enzyme Regulated Genes
Source: Int J Mol Sci. 2022 Sep 26;23(19):11322. doi: 10.3390/ijms231911322 (PMC9570307; doi:10.3390/ijms231911322)
Supplement: Supplementary file 1 [file ijms-23-11322-s001.zip › ijms-1890413-supplementary.pdf]

SUPPLEMENTARY MATERIALS AND METHODS

Generation of concentrated conditioned media* and *LOX Amplex Red Activity Assays.

Conditioned medium was concentrated essentially as previously described [1] with the following changes: Cells were seeded at ~80% confluency in 10 cm tissue culture plates. After ~12 hours cells were washed with PBS before addition of 10ml serum-free and phenol red-free DMEM supplemented with L-glutamine, gentamycin sulfate, amphotericin B, $CuSO_4$ (10 μM for HEK293 cells and 5 μM for MDA-MB-231 cells) and 0.1% BSA [1]. After 48 hours the conditioned medium was collected and concentrated 20 fold using 10k centrifugal filters. The resulted concentrated conditioned medium was used for LOX activity assays or immunoblotting. The LOX activity assay was also performed as previously described [1], with the following changes: Two separate solutions were made; the S solution containing Amplex® Red Reagent (A12222, Invitrogen) and DAP (1,5-diaminopentane, Cadaverin 95%, D22606-5G, Sigma. Acting as the substrate for LOX) in Assay Buffer (50 mM sodium borate, pH 8.0), and the E solution containing horseradish peroxidase in Assay Buffer. Solution E (50 μL) was added to 10μL of the LOX containing concentrated conditioned medium in All-black 96-well plates (Costar, Corning Inc). The irreversible LOX inhibitor -aminoproprionitrile (BAPN) [2] was then added to the control wells. Following addition of 40 μL of the S solution the plate was loaded into a FLUOstar Galaxy Multi-functional Microplate Reader (BMG Labtechnologies). Final concentrations of all reagents are as described in [1]. A kinetic top read of fluorescence was performed every 1 minute for a total of 179 minutes (excitation of 540 nm and emission 580 nm). Assays were done in duplicates. Activity was determined as the difference between the excitation and emission after 179 min of wells that contained or did not contain BAPN.

A

WT hLOXL2	Ala	Pro	Ala	Asn	Val	Ala	Lys	Ile	
	49	50	51	52	53	54	55	56	
#11 allele	Ala	Pro	Ala	Gln	Arg	Gly	Gln	Asp	Insertion +1
Only one chromosome / two identical									
WT hLOXL3	Arg	Val	Glu	Ile	Gln	Arg	Ala	Gly	
	57	58	59	60	61	62	63	64	
#1 allele 1	Arg	Val	Glu	Ilu	Gln	Arg	Ter*		Deletion -5
#1 allele 2	Arg	Val	His	Leu	Arg	Ter*			Deletion -29
WT hLOXL4	Lys	Tyr	Gly	Gln	Gly	Glu	Gly	Pro	
	87	88	89	90	91	92	93	94	
#1 allele 1	Lys	Tyr	Gly	Ser	Arg	Gly	Gly	Ter*	Insertion +1
#1 allele 2	Lys	Tyr	Ala	Arg	Gly	Gly	Ter*		Deletion -2
WT hLOX	Ala	Ser	Arg	Ala	Glu	Asn	Gln	Thr	
	139	140	141	142	143	144	145	146	
#28 allele 1	Ala	Ser	Arg	Arg	Gly	Glu	Pro	Asp	Insertion +1
#28 allele 2	Ala	Ser	Arg	Arg	Arg	Thr	Arg	Gln	Deletion -1
WT hLOXL1	Ser	Glu	Tyr	Val	Pro	Ala	Gly	Pro	
	59	60	61	62	63	64	65	66	
#6 allele 1	Ser	Glu	Tyr	Ala	Gly	Arg	Thr	Ser	Deletion -2
#6 allele 2	Ser	Glu	Tyr	Cys	Arg	Pro	Asp	Leu	Deletion -1

B

WT hLOX	Ala	Ser	Arg	Ala	Glu	Asn	Gln	Thr	
	139	140	141	142	143	144	145	146	
#17 allele 1	Ala	Ser	Arg	Arg	Gly	Glu	Pro	Asp	Insertion +1
#17 allele 2	Ala	Ser	Arg	Arg	Gln	Arg	Arg	Glu	Deletion -10

FIGURE S1

The effects of the frame shift mutations introduced into the alleles of the five lysyl-oxidases genes of the clone-6 5x knock-out cells and of 1x clone 17 knock-out cells on the amino-acid sequences immediately adjacent to the frame shift mutation: A. *The amino-acid sequence of the various wild type (WT) lysyl-oxidases near the location of the frame shift mutations is shown on top for each lysyl-oxidase. Underneath are indicated the predicted changes in the amino-acid sequences in each of the alleles of the lysyl-oxidase genes that were caused by the frame shift mutations. Changes in amino-acid composition are shown in red.* ***B.*** *The amino acid changes introduced into the proteins encoded by each of the alleles of the LOX gene in clone 17 knock-out cells. Changed amino-acids are in red.*

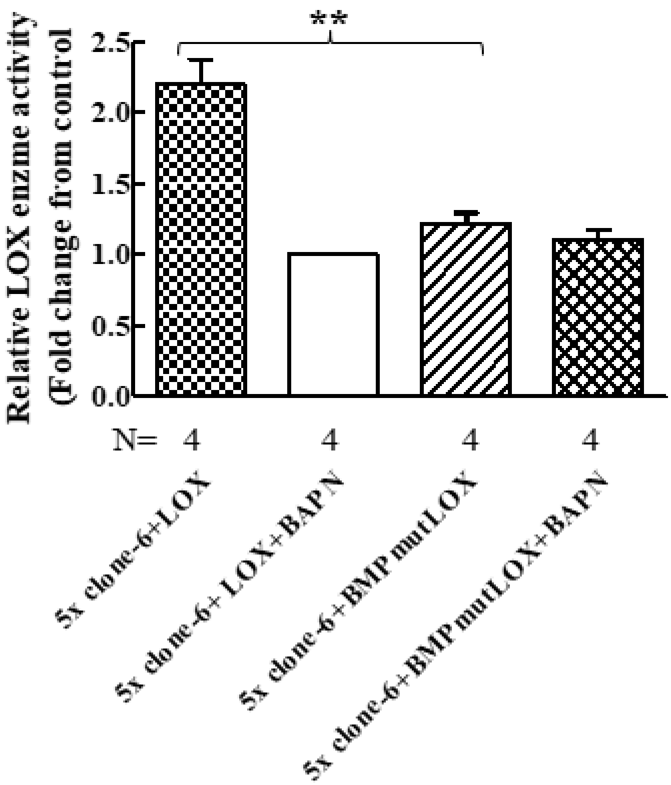

FIGURE S2

Changes in the amino-acid sequences of the lysyl-oxidases in clone-6 5x knock-out cells: The enzyme activity of the BMPmut LOX is inhibited: *LOX enzyme activity was determined in concentrated conditioned medium derived from the indicated cell lines in the presence or absence of BAPN. Shown is the mean excitation/emission ration at 179min of each sample. All sample ratios are normalized in reference to clone 6+LOX+BAPN. Statistical significance was evaluated using unpaired student's T-test with Welch's correction.*** $p < 0.01$.*

Table S1. sgRNA sequences used for CRISPR/Cas9 mediated knock-out of LOX family genes.

Gene	Top	Bottom
LOX (1)	CACCgTGTCTGGTTCTCCGCGCGCG	AAACCGCGCGCGGAGAACCAGACAc
LOX (2)	CACCgACGCGGACGGCCGGCTCATC	AAACGATGAGCCGGCCGTCCGCGTc
Loxl2	CACCgAATCTTGGCCACGTTGGCGG	AAACCCGCCAACGTGGCCAAGATTc
Loxl3	CACCgCGCGTGGAGATACAGCGAGC	AAACGCTCGCTGTATCTCCACGCGc
Loxl4	CACCgACAGTGCCAAGTACGGCCAA	AAACTTGGCCGTACTTGGCACTGTc
Loxl1	CACCgCTCGGGCTCAGAGTACGTGC	AAACGCACGTACTCTGAGCCCGAGc

Table S2. Primers used for construction of expression vectors directing expression of recombinant LOX and loxl genes using the NEBuilder HiFi DNA Assembly kits.

Primer description	Primer sequence
5' for inserting hloxl2 into NSPI	tgtggtggaattctgcagatatggagaggcctctgtgctc
3' for inserting hloxl2 into NSPI	cggccgccactgtgctggatttactgcggggacagctggt
5' for inserting hloxl3 into NSPI	tccagcacagtggcggccgcatgcgacctgtcagtgtctg
3' for inserting hloxl3 into NSPI	gttcgaagggccctctagacttagataatctggttgctggtctgg
5' for inserting hloxl4 into pENTR1A	cgactggatctcgagctcaatggcgtggtccccaccagc
3' for inserting hloxl4 into pENTR1A	ggtctagatatctcgagtgctcagatgaggttgttcctga
5' for inserting hLOX into NSPI (was also used for inserting hLOX, and	tgtggtggaattctgcagatatgcgcttcgcctggaccgt

the 1/3 part of LOXmut, BMPmutLOX and ADAmutLOX into NSPI with a MycHis tag)	
3' for inserting hLOX into NSPI	cggccgccactgtgctggatctaatacggtgaaattgtgc
3' for inserting the 1/3 part of LOXmut, BMPmutLOX and ADAmutLOX into NSPI with a MycHis tag	gctgtctggttctccgcgcgcgaggc
5' for inserting the 2/3 part of LOXmut, BMPmutLOX and ADAmutLOX into NSPI with a MycHis tag	gcctcgcgcgcggagaaccagacagc
3' for inserting the 2/3 part of LOXmut, BMPmutLOX and ADAmutLOX into NSPI with a MycHis tag	ataatctctgacatctgccctgtat
5' for inserting the 3/3 part of LOXmut, BMPmutLOX and ADAmutLOX into NSPI with a MycHis tag	atacagggcagatgtcagagattat
3' for inserting hLOX, and the 3/3 part of LOXmut, BMPmutLOX and ADAmutLOX into NSPI with a MycHis tag	cggccgccactgtgctggattatacggtgaaattgtgcag

Table S3. Sequences of the Taq-Man primers used for Quantitative real-time PCR analyses.

LOX	Hs00942480

loxl2	Hs00158757
loxl3	Hs01046945
loxl4	Hs00260059
STOX2	Hs01391114
AGR2	Hs00356521
DNAB11	Hs00212527
DNAJC3	Hs00939346
HSP90B1	Hs00427665
DERL3	HS00992980
HSPA5	HS00607129
RPLPO	Hs99999902

REFERENCES

1. Hutchinson, J. H.; Rowbottom,M.W.; Lonergan,D.; Darlington,J.; Prodanovich,P.; King,C.D.; Evans,J.F.; Bain,G. Small Molecule Lysyl Oxidase-like 2 (LOXL2) Inhibitors: The Identification of an Inhibitor Selective for LOXL2 over LOX. *ACS Med. Chem. Lett.* 2017 **8,** 423-427

2. Tang, S. S.; Trackman,P.C.; Kagan,H.M. Reaction of aortic lysyl oxidase with beta-aminopropionitrile. *J. Biol. Chem.* 1983 **258,** 4331-4338
